# Lack of Neuronal Glycogen Impairs Memory Formation and Learning-Dependent Synaptic Plasticity in Mice

**DOI:** 10.3389/fncel.2019.00374

**Published:** 2019-08-13

**Authors:** Jordi Duran, Agnès Gruart, Olga Varea, Iliana López-Soldado, José M. Delgado-García, Joan J. Guinovart

**Affiliations:** ^1^Institute for Research in Biomedicine (IRB Barcelona), The Barcelona Institute of Science and Technology, Barcelona, Spain; ^2^Centro de Investigación Biomédica en Red de Diabetes y Enfermedades Metabólicas Asociadas (CIBERDEM), Madrid, Spain; ^3^Division of Neurosciences, Pablo de Olavide University, Seville, Spain; ^4^Department of Biochemistry and Molecular Biomedicine, University of Barcelona, Barcelona, Spain

**Keywords:** glycogen, learning, LTP, memory, metabolism

## Abstract

Since brain glycogen is stored mainly in astrocytes, the role of this polysaccharide in neurons has been largely overlooked. To study the existence and relevance of an active neuronal glycogen metabolism *in vivo*, we generated a mouse model lacking glycogen synthase specifically in the Camk2a-expressing postnatal forebrain pyramidal neurons (GYS1^Camk2a–KO^), which include the prefrontal cortex and the CA3 and CA1 cell layers of the hippocampus. The latter are involved in memory and learning processes and participate in the hippocampal CA3-CA1 synapse, the function of which can be analyzed electrophysiologically. Long-term potentiation evoked in the hippocampal CA3-CA1 synapse was decreased in alert behaving GYS1^Camk2a–KO^ mice. They also showed a significant deficiency in the acquisition of an instrumental learning task – a type of associative learning involving prefrontal and hippocampal circuits. Interestingly, GYS1^Camk2a–KO^ animals did not show the greater susceptibility to hippocampal seizures and myoclonus observed in animals completely depleted of glycogen in the whole CNS. These results unequivocally demonstrate the presence of an active glycogen metabolism in neurons *in vivo* and reveal a key role of neuronal glycogen in the proper acquisition of new motor and cognitive abilities, and in the changes in synaptic strength underlying such acquisition.

## Introduction

Glycogen is a polymer of glucose that is synthesized by glycogen synthase (GS), and it acts as an energy reservoir. In the brain, it is present at concentrations much lower than those found in muscle or liver. It has traditionally been accepted that brain glycogen is stored exclusively in astrocytes. However, an increasing number of observations reveal the presence of an active glycogen metabolism in neurons. Indirect evidence comes from conditions such as Lafora disease and Pompe disease, in which glycogen abnormally accumulates in this cell population (Sidman et al., 2008; Duran et al., 2014). Furthermore, we previously demonstrated that primary cultured neurons have an active glycogen metabolism that protects them from hypoxia-induced death (Saez et al., 2014).

The use of genetically modified animals has allowed the study of the normal and pathological aspects of brain glycogen. The capacity to delete specific genes only in the brain ensures that the results reflect the effects of brain glycogen, without the influence of the manipulation of glycogen metabolism in other organs. In this regard, we generated a mouse model devoid of GS (and thus of glycogen) in the CNS (GYS1^Nestin–KO^) (Duran et al., 2013). These animals showed a significant deficit in learning capacity, which unequivocally demonstrated the key role of brain glycogen in the proper acquisition of relatively difficult learning tasks, in accordance with previous results obtained using inhibitors of glycogen degradation (Gibbs et al., 2006; Suzuki et al., 2011). We also described changes in synaptic strength at the electrophysiological level that correlate with this deficit in learning capacity. Furthermore, GYS1^Nestin–KO^ mice showed greater susceptibility to hippocampal seizures and myoclonus following the administration of kainate and/or a brief train stimulation of Schaffer collaterals. Thus, these observations indicate that brain glycogen plays a protective role in the prevention of brain seizures (Lopez-Ramos et al., 2015).

However, since GS was ablated from both astrocytes and neurons in these animals, it was not possible to dissect whether these abnormalities were due to the lack of astrocytic or neuronal glycogen. Therefore, to address the existence and relevance of an active glycogen metabolism in neurons *in vivo*, we generated a new mouse model lacking GS specifically in the Camk2a-positive neurons of the forebrain, including the prefrontal cortex and hippocampal pyramidal cells involved in CA3-CA1 synapses (GYS1^Camk2a–KO^).

These animals showed decreased long-term potentiation (LTP) evoked in the hippocampal CA3-CA1 synapse and a significant deficiency in the acquisition of an instrumental learning task – a type of associative learning involving both prefrontal and hippocampal circuits. In contrast, they did not show differences in a fear-conditioning task, which involves medial prefrontal-amygdala circuits. Furthermore, these mice did not present the greater susceptibility to hippocampal seizures and myoclonus observed in the GYS1^Nestin–KO^ model.

These results unequivocally demonstrate the presence of an active glycogen metabolism in neurons *in vivo* and its fundamental role in the proper acquisition of new motor and cognitive abilities and in the changes in synaptic strength underlying such acquisition.

## Materials and Methods

### Animals

Experiments were carried out in C57BL/6 male mice aged 5.4 ± 1.3 months (GYS1^Camk2a–KO^) and 5.1 ± 1.7 months (control littermates) (*n* = 15 for each group). All experiments were carried out following Spanish (BOE 34/11370-421, 2013) and European Union (2010/63/EU) regulations on the use of laboratory animals. All experimental protocols were approved by the Ethics Committee of the Pablo de Olavide University. Animals were kept in collective cages (up to five animals per cage) on a 12-h light/dark cycle with constant temperature (21 ± 1°C) and humidity (50 ± 5%) until the beginning of the experiments. Afterward, they were kept in individual cages until the end of the study. Unless otherwise indicated, animals were allowed access *ad libitum* to commercial mouse chow and water.

### Biochemical Analyses

Animals were anesthetized, perfused intracardiacally with saline, and killed by decapitation. Brains were removed and cortexes, hippocampi and cerebella were dissected under a loupe, frozen on liquid nitrogen and stored at **–**80°C until use. For western blot analyses, protein homogenates of the different regions were obtained using the following buffer: 25 mM Tris–HCl (pH 7.4), 25 mM NaCl, 1% Triton X-100, 0.1% SDS, 0.5 mM EGTA, 10 mM sodium pyrophosphate, 1 mM sodium orthovanadate, 10 mM NaF, 25 nM okadaic acid and a protease inhibitor cocktail tablet (Roche). Homogenates were loaded in 10% acrylamide gels for SDS-PAGE and transferred to Immobilon membranes (Millipore). The antibody against GYS1 used was ref. 3886 from Cell Signaling. Proteins were detected by the ECL method (Immobilon Western Chemiluminescent HRP Substrate, Millipore) and loading control of the western blot membrane was performed using the REVERT total protein stain. For quantitative (q)PCR, total RNA was isolated from frozen brain region samples using Trizol reagent (Life Technologies, Carlsbad, CA, United States), purified with an RNeasy Mini Kit (Qiagen, Hilden, Germany) and treated with DNase I (Qiagen) to degrade genomic DNA. Reverse transcription was performed using qScript cDNA Synthesis Kit (Quanta Biosciences, Beverly, MA, United States). qPCR was performed using a Quantstudio 6 Flex (Applied Biosystems, Foster City, CA, United States). The following mouse-specific SYBRgreen set of primers (Sigma, Madrid, Spain) was used: Gys1 (forward: 5′-CAGAGCAAAGCACGAATCCA-3′; reverse: 5′-CATAGCG GCCAGCGATAAAG-3′); Gys2 (forward: 5′-ACCAAGGCC AAAACGACAG-3′; reverse: 5′-GGGCTCACATTGTTCTACT TGA-3′) and beta-2 microglobulin (b2M), used as a housekeeping gene (forward: 5′-ATGCACGCAGAAAG AAATAGCAA-3′; reverse: 5′-AGCTATCTAGGATATTTCC AATTTTTGAA-3′). All the samples were run as triplicates. For representation of the results: dCt was calculated as: Ct (b2M)-Ct (gene of interest), and average dCt from control hippocampus was used to calculate ddCT. Results are expressed as 2^ddCt in relative units for each brain region and genotype analyzed.

### Operant Conditioning

Following previous descriptions by some of the authors of this paper (Madronal et al., 2010; Jurado-Parras et al., 2012), training and testing was done in three Skinner box operant chambers (12.5 × 13.5 × 18.5 cm) (MED Associates, St. Albans, VT, United States). Each module was housed within a sound-attenuating chamber (90 × 55 × 60 cm), which was constantly lit (19 W lamp) and exposed to 45-dB white noise (Cibertec, S.A., Madrid, Spain). Each Skinner box was equipped with a food dispenser from which pellets (MLabRodent Tablet, 20 mg; Test Diet, Richmond, IN, United States) were released by pressing a lever. Before training, mice were handled daily for 5–7 days and food-deprived to 90% of their free-feeding weight. Training took place for 20 min on successive days, in which mice were trained to press the lever to receive pellets from the food dispenser using a fixed-ratio (1:1) schedule. The start and end of each session was indicated by a tone (2 kHz, 200 ms, 70 dB) provided by a loudspeaker located in the recording chamber. Animals were maintained on this fixed-ratio (1:1) schedule until they reached the selected criterion, namely until they obtained ≥ 20 pellets in two successive sessions. The mean number of pellets received by each animal during these two sessions was calculated.

In the following experimental step, conditioning was carried out for 10 days using a light/dark protocol. In this protocol, only lever presses performed by the animal during the light period (20 s), in which a small light bulb located over the lever was switched on, were reinforced with a pellet. Lever presses performed during the dark period (20 ± 10 s) were not rewarded. In addition, lever presses during the dark period restarted the dark protocol for an additional random (1–10 s) time. The number of lever presses carried out during the light and dark periods were counted. The light/dark coefficient was calculated as follows: (number of lever presses during the light period − number of lever presses during the dark period)/total number of lever presses. As we previously described, control mice never obtain positive coefficients > 0.2 in this task (Hasan et al., 2013). Conditioning programs, lever presses, and pellets rewarded were monitored and recorded by a computer, using a MED-PC program (MED Associates, St. Albans, VT, United States). All operant sessions were filmed with a synchronized video capture system (Sony HDR-SR12E, Tokyo, Japan).

### Surgery

A minimum of a week after the end of operant conditioning experiments, animals were anesthetized with 0.8–3% halothane delivered from a calibrated Fluotec 5 (Fluotec-Ohmeda, Tewksbury, MA, United States) vaporizer at a flow rate of 1–2 L/min oxygen. Animals were implanted with bipolar stimulating electrodes at the right Schaffer collateral-commissural pathway of the dorsal hippocampus (2 mm lateral and 1.5 mm posterior to bregma; depth from brain surface, 1.3 mm) (Paxinos and Franklin, 2001) and with two recording electrodes in the ipsilateral CA1 area (1.2 mm lateral and 2.2 mm posterior to bregma; depth from brain surface, 1.3 mm). They were also implanted bilaterally with two pairs of recording electrodes in the prelimbic cortex (0.3 mm lateral, 1.75 mm anterior to bregma, and 1.8 from brain surface) (Paxinos and Franklin, 2001). Electrodes were made of 50 μm Teflon-coated tungsten wire (Advent Research Materials Ltd., Eynsham, England). The final location of the CA1 recording electrode was determined using as a guide the field potential depth profile evoked by paired (40 ms of interval) pulses presented at the Schaffer collateral pathway. Two bare silver wires (0.1 mm) were affixed to the skull as ground. The 10 wires were connected to a 4-pin and a 6-pin socket. Sockets were fixed to the skull with the help of small screws and dental cement (see Gruart et al., 2006 for details).

### Fear Conditioning

Fear conditioning was carried out as previously described (Ortiz et al., 2010; Madronal et al., 2016). A week after surgery, animals were subjected to a fear conditioning test. On the first training day, they were subjected to a three-shock acquisition protocol in a fear-conditioning box (25 × 25 × 35 cm; TSE Systems GmbH, Bad Homburg, Germany) provided with a metallic grid floor, transparent Plexiglas wall, 70% ethanol smell, and 800 lux illumination (context A). Mice were allowed to explore the box for 3 min, after which a 30-s, 80-dB, 7.5-kHz tone was given as a conditioning stimulus (CS). The last 2 s of the tone overlapped with a 0.4-mA foot shock as unconditioned stimulus (US). The CS-US pairing was repeated two more times with 2-min intervals between each presentation and following the last one. Therefore, the fear conditioning acquisition test lasted for 10.5 min. Two days after the test session, animals were placed in the same box for 6 min but no CS or US was given. Finally, the cued fear test was repeated 3 days later in the same fear-conditioning box but this time with different floor (opaque gray plastic) and walls (black plastic), including acetic acid smell and 400 lux illumination (context B). Mice were allowed to explore the box for 3 min, after which the CS was presented continuously for another 3 min (total duration 6 min).

### Input/Output Curves, Paired Pulse Facilitation, and LTP in Behaving Mice

Local field potentials (LFPs) were recorded with the animal located in a small (5 × 5 × 5 cm) box, aimed to avoid overwalking (Gruart et al., 2006). For input/output curves, mice were stimulated at the CA3-CA1 synapse with single pulses at increasing intensities (0.02–0.4 mA). We also checked the effects of paired pulses at various (10, 20, 40, 100, 200, and 500 ms) inter-pulse intervals, using intensities corresponding to ∼ 40% of the amount necessary to evoke a saturating response. In all cases, pulses of a given intensity and/or interval were repeated 10 times at a rate of 3/min, to avoid interferences with slower short-term potentiation (augmentation) or depression processes (Zucker and Regehr, 2002); moreover, to avoid any cumulative effect, intensities and/or intervals were presented at random.

To evoke LTP in behaving mice, we followed procedures described previously (Gruart et al., 2006). Baseline field excitatory postsynaptic potential (fEPSP) values evoked at the CA3-CA1 synapse were collected 15 min prior to LTP induction using single 100 μs, square, biphasic pulses. Pulse intensity was set at ∼40% of the amount necessary to evoke a maximum fEPSP response (0.15–0.25 mA) – i.e., well below the threshold for causing a population spike. For LTP induction, animals were presented with a high-frequency stimulus (HFS) protocol consisting of five 200 Hz, 100-ms trains of pulses at a rate of 1/s, repeated six times, at intervals of 1 min. Thus, a total of 600 pulses were presented during the HFS session. To avoid evoking large population spikes and/or electroencephalographic seizures, the stimulus intensity during HFS was set at the same value as that used for generating baseline recordings. After each HFS session, the same stimuli were presented individually every 20 s for 60 additional min and for 30 min on the following 4 days.

### Hippocampal Seizures Evoked by Kainate Injection

In order to determine the propensity of control and GYS1^Camk2a–KO^ mice to generate convulsive seizures, animals were injected (i.p.) with the AMPA/kainate receptor agonist kainic acid (8 mg/kg; Sigma, St. Louis, MO, United States) dissolved in 0.1 M phosphate buffered saline (PBS) pH = 7.4. LFPs were recorded in the hippocampal CA1 area from 5 min before to 60 min after kainate injections (see Valles-Ortega et al., 2011 for details).

### Histology

Once the experiments had ended, mice were deeply re-anesthetized (sodium pentobarbital, 50 mg/kg) and perfused transcardially with saline and 4% phosphate-buffered paraformaldehyde. Their brains were removed, postfixed overnight at 4°C, and cryoprotected in 30% sucrose in PBS. Sections were obtained in a microtome (Leica, Wetzlar, Germany) at 50 μm. Selected sections, including the dorsal hippocampus and the prefrontal cortex, were mounted on gelatinized glass slides and stained with 0.1% toluidine blue to determine the location of stimulating and recording electrodes.

### Experimental Design and Statistical Analysis

fEPSPs, 1-V rectangular pulses corresponding to lever presses, pellet delivery, tone and shock presentations, and brain stimulation were stored digitally on a computer through an analog/digital converter (CED 1401 Plus, CED, Cambridge, England). Recorded videos were synchronized to the CED recording system. Data were analyzed off-line for quantification of each animal’s performance in the Skinner box, in the fear conditioning test, and for fEPSP recordings with the Spike 2 (CED) program. The slope of evoked fEPSPs was computed as the first derivative (V/s) of fEPSP recordings (volts). Five successive fEPSPs were averaged, and the mean value of the slope during the rise-time (i.e., the period of the slope between the initial 10% and the final 10% of the fEPSP) was determined. The power spectrum of recorded LFPs activity was computed with the help of the Mat Lab 7.4.0 software (MathWorks, Natick, MA, United States), using the fast Fourier transform with a Hanning window, expressed as relative power and averaged across each recording session (Munera et al., 2000). Computed results were processed for statistical analysis using the IBM SPSS Statistics 18.0 (IBM, Armonk, NY, United States). Data are represented as the mean ± SEM. Statistical significance of differences between groups was inferred by one-way ANOVA and ANOVA for repeated measures (data by groups), with a contrast analysis (Dunnett’s posttest) for a further study of significant differences. Statistical significance was set at *p* < 0.05.

## Results

### Generation of Neuron-Specific GYS1^Camk2a–KO^ Animals

**FIGURE 1:**
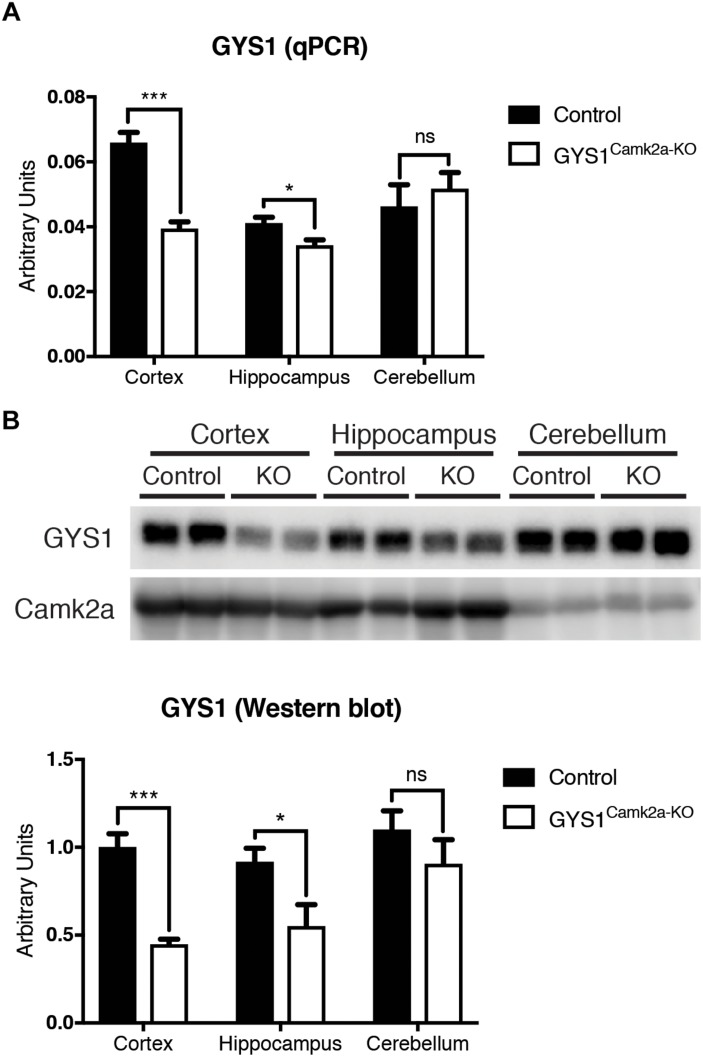
Analysis of GYS1 expression in brain regions of control and GYS1^Camk2a–KO^ mice. **(A)** qPCR analysis of GYS1 gene expression. The expression of GYS1 was measured in the cortex, hippocampus and cerebellum of control (*n* = 4) and GYS1^Camk2a–KO^ mice (*n* = 5). The data shown are mean ± SEM of 2^ddCt in relative units for each genotype analyzed. Significant differences between groups were found in the cortex (*p* < 0.0001) and hippocampus (*p* = 0.0383), but not in cerebellum. **(B)** Western blot analyses of GYS1 protein and quantification of the results. Protein homogenates of the same regions were analyzed by western blot with an antibody specific for GYS1 protein (*n* = 4 per region and genotype). Again significant differences between groups were found in the cortex (*p* = 0.0006) and hippocampus (*p* = 0.0474), but not in the cerebellum. Statistics were calculated using unpaired *t*-test, statistical values. ^*^p < 0.05, ^∗∗∗^*p* < 0.001.

GYS1^Camk2a–KO^ animals were generated by crossing GYS1 conditional knockout, based on the Cre/lox technology (Duran et al., 2013), with Camk2a Cre animals, which express Cre recombinase specifically in Camk2a-positive neurons of the forebrain (Tsien et al., 1996). Homozygous conditional non-expressing Cre littermates were used as controls. To corroborate the efficiency of the deletion, we analyzed the expression of GYS1 by qPCR and western blot in three regions of the brain, namely the hippocampus, cortex and cerebellum. qPCR results showed a significantly lower expression of GYS1 in hippocampus and cortex of GYS1^Camk2a–KO^ mice. As expected, no differences in GYS1 expression were found between the cerebella of the two genotypes, since the Camk2a-cre mouse line does not have a significant Cre recombinase expression in this region (Tsien et al., 1996) (Figure 1A). The difference between control and GYS1^Camk2a–KO^ mice was greater in the cortex than in the hippocampus, probably due to the higher ratio of astrocytes to neurons present in the latter (Keller et al., 2018), which would dilute the difference in GYS1 expression that results from knocking out only Camk2a neurons. To discard a compensatory upregulation, GYS2 expression levels were also measured. However, no significant expression in any of the regions in either genotype was detected (data not shown). At the protein level, western blot analyses also showed a significant difference in GYS1 in the cortex and hippocampus, but not in the cerebellum (Figure 1B).

### Differences in the Electrophysiological Properties of the CA3-CA1 Synapse in Control and GYS1^Camk2a–KO^ Mice

**FIGURE 2:**
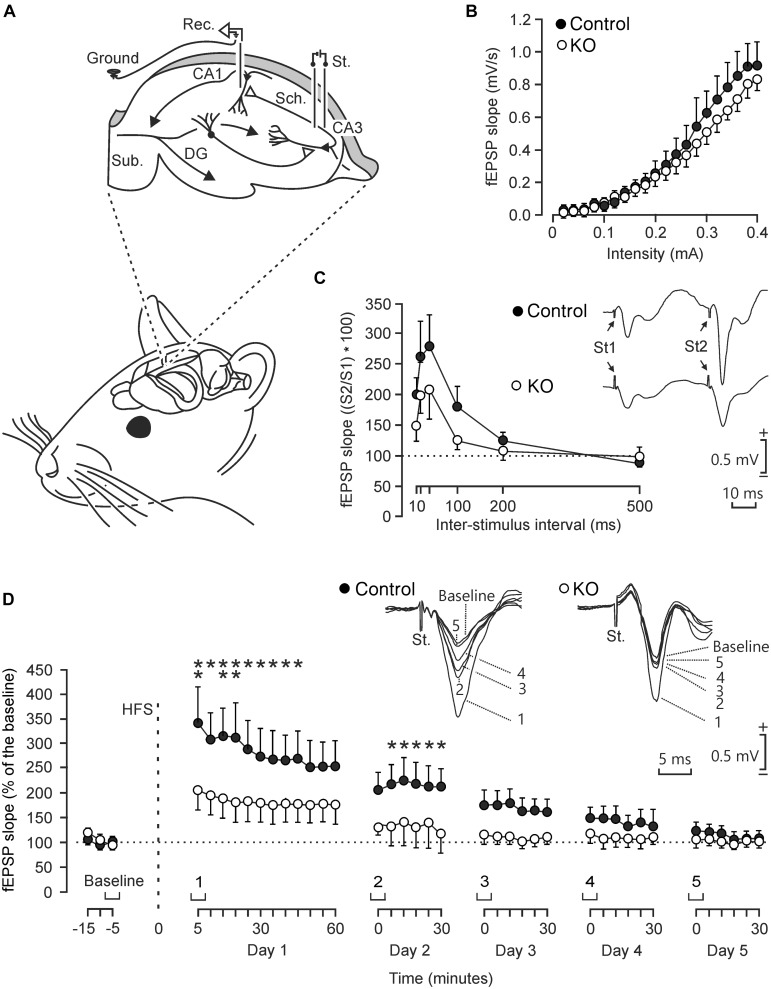
Electrophysiological properties of hippocampal synapses in alert behaving control and GYS1^Camk2a–KO^ mice. **(A)** Experimental design. Animals were chronically implanted with stimulating electrodes in CA3 Schaffer collaterals and with a recording electrode in the ipsilateral CA1 area. An extra wire was attached to the bone as ground. DG, dentate gyrus; Sub., subiculum. Adapted from Duran et al. (2013). **(B)** Input/output curves of fEPSPs evoked at the CA3-CA1 synapse by paired (40 ms of inter-pulse interval) pulses of increasing intensities (0.02–0.4 mA) in control (*n* = 8; black circles) and GYS1^Camk2a–KO^ (*n* = 8; white circles) mice. Data are represented as mean ± SEM. No significant differences [*F*_(__19__,__266__)_ = 0.579; *p* = 0.92] were observed between groups. **(C)** In addition, no significant [*F*_(__5__,__110__)_ = 0.753; *p* = 0.586] differences in paired-pulse facilitation between control (*n* = 12; black circles) and GYS1^Camk2a–KO^ (*n* = 12; white circles) mice were detected. The data shown are mean ± SEM slopes of the 2nd fEPSP expressed as a percentage of the first of six (10, 20, 40, 100, 200, 500) inter-pulse intervals. Selected fEPSP paired traces (40 ms of inter-pulse interval) collected from representative control and GYS1^Camk2a–KO^ mice are shown at the top right. **(D)** The two graphs illustrate the time course of LTP evoked in the CA3-CA1 synapse (fEPSP mean ± SEM) of control (*n* = 14; black circles) and GYS1^Camk2a–KO^ (*n* = 14; white circles) mice following a HFS session. The HFS was presented after 15 min of baseline recordings, at the time marked by the dashed line. LTP evolution was monitored over 5 days. At the top, illustrated representative examples of fEPSPs from representative control and GYS1^Camk2a–KO^ mice collected at the times indicated in the bottom graphs. fEPSP slopes are given as a percentage of fEPSP values collected during baseline recordings (100%). Although the two groups presented significant (*p* < 0.001) increases (ANOVA, two-tailed) in fEPSP slopes after HFS when compared with baseline recordings, the control group showed a larger and longer lasting LTP [*F*_(__38__,__988__)_ = 2.049; *p* < 0.001] than GYS1^Camk2a–KO^ mice. fEPSP slopes collected from control animals were significantly larger than those from GYS1^Camk2a–KO^ mice at the indicated times (^*^p < 0.05; ^∗∗^*p* < 0.01).

To determine functional differences in the electrophysiological properties of hippocampal circuits, we studied input/output curves, paired-pulse facilitation, and LTP evoked at the CA3-CA1 synapse in behaving control and GYS1^Camk2a–KO^ mice (Figure 2). First, we examined the response of CA1 pyramidal neurons to paired-pulses (40 ms of inter-pulse intervals) of increasing intensity (0.02–04 mA) presented to the ipsilateral Schaffer collaterals (Figure 2A). Control and GYS1^Camk2a–KO^ mice presented similar increases in the slope of fEPSP evoked at the CA3-CA1 synapse by the stimuli presented to the CA3 area (Figure 2B). These two relationships were best fitted by sigmoid curves (*r* ≥ 0.99; *p* < 0.0001; not illustrated), thereby suggesting the normal functioning of the CA3-CA1 synapse in both groups.

The paired-pulse facilitation evoked in control and GYS1^Camk2a–KO^ mice was then analyzed with increasing inter-pulse intervals (10, 20, 40, 100, 200, 500 ms), but presenting a fix stimulus intensity (40% of asymptotic values). Both groups of mice presented a paired pulse facilitation at short (20 and 40 ms) inter-pulse intervals (Figure 2C). Although control mice presented slightly larger facilitation to paired pulses than GYS1^Camk2a–KO^ animals, no significant differences were observed.

We next studied LTP in the two groups of behaving mice as a reflection of synaptic plasticity. The CA3-CA1 synapse is involved in the acquisition of various kinds of associative learning tasks and is usually selected for evoking LTP in behaving mice (Gruart et al., 2006). For baseline values, animals were stimulated every 20 s for ≥15 min at the implanted Schaffer collaterals (Figure 2D). Afterward, they were presented with the high frequency stimulation (HFS) protocol. Following HFS, the same single stimulus used to generate baseline records was presented at the initial rate (3/min) for another 60 min. Recording sessions were repeated for four additional days (30 min each; Figure 2D). Both groups of mice presented a significant increase in fEPSP slopes following the HFS session. However, the control group presented a larger and longer lasting LTP than the GYS1^Camk2a–KO^ group. The point to point comparison between LTPs evoked in the two groups of mice indicated that WT animals presented larger LTP values for the five recording sessions and that these values were significantly different during the first two sessions (*p* ≤ 0.05) (Figure 2D).

In summary, at the electrophysiological level, GYS1^Camk2a–KO^ mice presented similar input/output curves and paired-pulse potentiation but less LTP than their littermate controls.

### GYS1^Camk2a–KO^ Mice Presented a Deficit in the Acquisition of an Instrumental Conditioning Task

**FIGURE 3:**
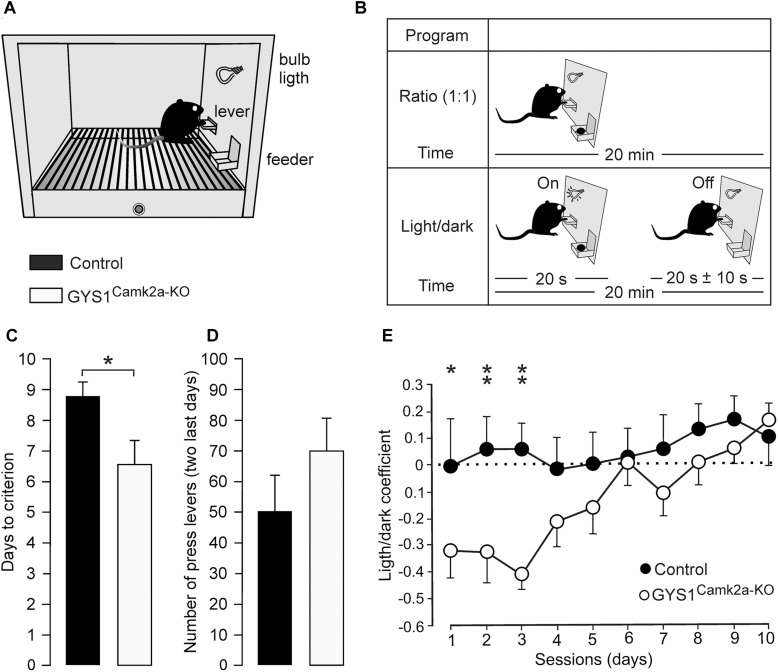
Performance of control and GYS1^Camk2a–KO^ mice in an operant conditioning task. **(A)** Experimental setup. Mice were trained in a Skinner box to press a lever to obtain a food pellet, with a fixed-ratio (1:1) schedule. Adapted from Duran et al. (2013). **(B)** Animals were trained with two programs of increasing difficulty. As illustrated in the top diagram, they were first trained to acquire a fixed-ratio (1:1) schedule until obtaining 20 pellets/20 min session on two successive days (criterion). Afterward, lever presses were rewarded only when a light bulb was switched on (see bottom diagram). **(C)** Time to reach the selected criterion for control (*n* = 13) and GYS1^Camk2a–KO^ (*n* = 15) mice (*t* = 239.000; *p* = 0.017; Shapiro–Wilk *t* test). **(D)** Data collected from the first 2 days after reaching the criterion with the fixed-ratio (1:1) schedule. Illustrated data correspond to the mean ± SEM collected from control (*n* = 13) and GYS1^Camk2a–KO^ (*n* = 15) animals. Note that although GYS1^Camk2a–KO^ mice pressed the lever more frequently than the control group, there were no significant differences between groups (*U* = 58.5; *p* = 0.13; Mann–Whitney *U* statistic test). **(E)** Performance of control (*n* = 13) and GYS1^Camk2a–KO^ (*n* = 14) mice during the light/dark test. Note that although both groups showed an improvement in performance across sessions [*F*_(__1__,__225__)_ = 10.49; *p* < 0.01], control mice outperformed GYS1^Camk2a–KO^ mice [*F*_(__9__,__225__)_ = 2.82; *p* = 0.01]. The light/dark coefficient was calculated as follows: (number of lever presses during the light period – number of lever presses during the dark period)/total number of lever presses. For individual sessions ^*^p = 0.05; ^∗∗^*p* = 0.01.

The acquisition of operant conditioning tasks involves many cortical (medial prefrontal and motor cortices, hippocampus) and subcortical (dorsal and ventral striatum) structures (Jurado-Parras et al., 2012, 2013). The capacity of the two groups of mice to acquire these complex associative learning tasks was examined. Animals were trained in Skinner box modules to obtain a food pellet every time they pressed a lever located near the feeder (Figure 3A). In a first series of experiments, we checked the time (days) animals needed to learn this operant task, using a fixed-ratio (1:1) schedule. The criterion was to press the lever a minimum of 20 times/session for two successive 20-min sessions (Figure 3B, top diagram). A total of 13 controls (out of 15) and 15 GYS1^Camk2a–KO^ (out of 15) mice reached the criterion for the fixed-ratio (1:1) schedule task in ≤10 days (8.8 ± 0.5 days for controls and 6.7 ± 0.7 days for KO; *t* = 239.000; *p* = 0.017; Shapiro–Wilk *t* test; Figure 3C). GYS1^Camk2a–KO^ mice seemed to press the lever more frequently than controls (Figure 3D), which was suggestive of a sort of compulsive behavior in GYS1^Camk2a–KO^. Animals from the two groups that reached the criterion for the fixed-ratio (1:1) schedule task in ≤10 days were further trained in a more complex task. In this case, they were rewarded [again in a fixed-ratio (1:1) schedule] only during the period in which a small light bulb, located over the lever, was switched on (Figure 3B, bottom diagram). Periods with light lasted for 20 s and were followed by dark periods during which the animal was not rewarded. Pressing the lever during the dark period punished the animal by delaying the reappearance of the light period by ≤10 additional seconds (see Jurado-Parras et al., 2012; Hasan et al., 2013). Control mice acquired this complex task earlier than GYS1^Camk2a–KO^ mice, reaching significant differences during the first three training sessions (Figure 3E). Indeed, GYS1^Camk2a–KO^ mice took longer (up to 5 sessions) to understand that pressing the lever during the dark period did not provide any reinforcement and, in addition, delayed the reappearance of light. In conclusion, the deficit in neuronal glycogen caused a significant impairment of the learning capacity of GYS1^Camk2a–KO^ mice, which was probably aggravated by deficient control of compulsive behaviors.

### GYS1^Camk2a–KO^ Mice Showed a Similar Performance to Control Animals in a Fear Conditioning Task

**FIGURE 4:**
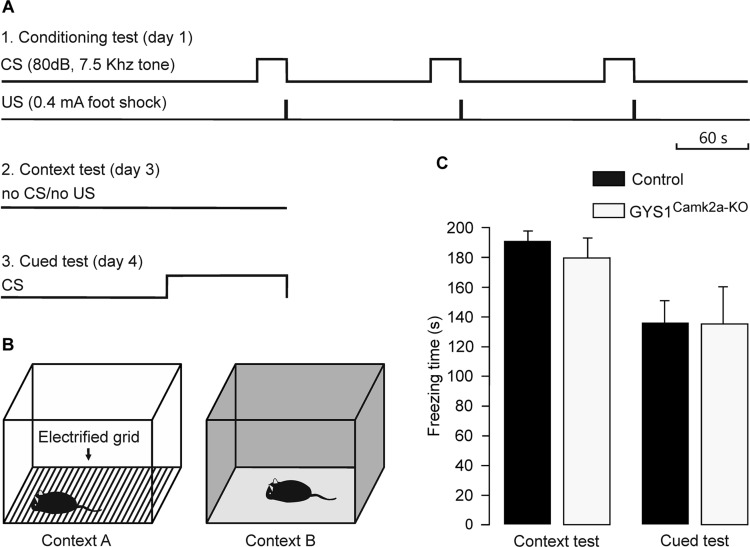
Performance of control and GYS1^Camk2a–KO^ mice in a fear conditioning task. **(A)** Experimental design. During the conditioning test (1), animals were allowed to explore context A for 3 min, after which they were presented with a conditioning tone for 30 s (CS). The tone co-terminated with a foot shock (0.4 mA, 2 s). As illustrated, the CS-US pairing was repeated two more times. For the context test (2), the animal was located in the same box for 6 min in absence of CS or US presentations. Finally, for the cued test (3), the animal was located in context B for a control period of 3 min, followed by 3 min with a CS presentation. **(B)** Diagrammatic representation of contexts A (left) and B (right). **(C)** No significant differences between the two groups (*n* = 14 control and *n* = 15 GYS1^Camk2a–KO^ mice) were observed for either of context (*t* = 0.595; 27 degrees of freedom; *p* = 0.557) or the cued tests (*t* = 0.0166; 27 degrees of freedom; *p* = 0.987).

Medial prefrontal-amygdala circuits are actively involved in the control of fear conditioning (Dejean et al., 2016). In particular, it has been shown that delta oscillations synchronize these circuits during fear conditioning (Karalis et al., 2016). Given these observations, we carried out a fear conditioning test in control (*n* = 14) and GYS1^Camk2a–KO^ (*n* = 15) mice (Figures 4A,B). Freezing time during the context test was similar for the two groups (control, 190.5 ± 9.0 s; GYS1^Camk2a–KO^, 182.5 ± 10.0 s). In addition, the total freezing time during the cued fear test was also similar for both groups (control, 136.7 ± 17.2 s; GYS1^Camk2a–KO^, 136.5 ± 22.7 s) (Figure 4C). According to these results, amygdala control of fear behavior (Dejean et al., 2016) was not altered in GYS1^Camk2a–KO^ mice.

### Susceptibility to Kainate-Induced Epilepsy in Control and GYS1^Camk2a–KO^ Mice

**FIGURE 5:**
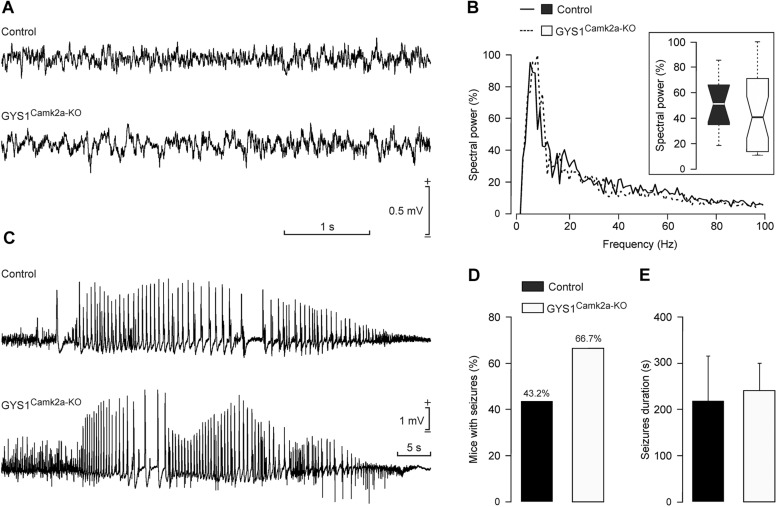
Susceptibility of control and GYS1^Camk2a–KO^ mice to kainate. **(A)** RLFPs recorded in the CA1 area of representative control and GYS1^Camk2a–KO^ mice. **(B)** Spectral power of LFPs (60-s segments collected from *n* = 12 control and *n* = 12 GYS1^Camk2a–KO^ mice). The inset illustrates the global boxplot of peak spectral powers [*F*_(__1__,__22__)_ = 0.07; *p* = 0.7908]. **(C)** Representative examples of hippocampal seizures evoked in control and GYS1^Camk2a–KO^ mice following the administration of 8 mg/kg i.p. of kainate. **(D)** Percentage of control (*n* = 13) and GYS1^Camk2a–KO^ (*n* = 15) mice presenting spontaneous seizures at the CA1 area during the recording period (60 min). No significant differences between groups (Chi-square = 0.506 with 1 degree of freedom; *p* = 0.477) were observed. **(E)** Mean duration of kainate-evoked seizures in control and GYS1^Camk2a–KO^ mice. No significant differences between groups were observed [*F*_(__1__,__14__)_ = 0.0604; *p* = 0.809; ANOVA and Shapiro–Wilk test].

Local field potentials recorded in the hippocampus of control (*n* = 12) and GYS1^Camk2a–KO^ (*n* = 12) mice presented no significant differences in their respective spectral powers (Figures 5A,B). In addition, we checked the susceptibility of the two groups to a single and low dose (8 mg/kg) i.p. injection of kainate (Figures 5C–E). Both control (6 out of 13; 43.2%) and GYS1^Camk2a–KO^ (10 out of 15; 66.7%) mice presented spontaneous hippocampal seizures after kainate injection, with no significant differences between groups (Chi-square = 0.506 with 1 degree of freedom; *p* = 0.477) (Figure 5D). The mean duration of kainate-evoked seizures was also similar between groups (control: 214 ± 111 s; GYS1^Camk2a–KO^: 242 ± 57 s; *F*_(__1__,__14__)_ = 0.0604; *p* = 0.809; ANOVA and Shapiro–Wilk test) (Figure 5E). These results indicate that susceptibility to kainate-induced epilepsy is not significantly altered in GYS1^Camk2a–KO^ mice with respect to littermate controls.

## Discussion

Brain glycogen is involved in multiple functions, including learning and memory. In this regard, in a previous study we showed that GYS1^Nestin–KO^ mice, which are devoid of glycogen in the brain, show a significant impairment of learning capacity and in the concomitant activity-dependent changes in synaptic strength (Duran et al., 2013). In a subsequent report with the same mouse model, we demonstrated the importance of brain glycogen in resistance to epileptic seizures (Lopez-Ramos et al., 2015). However, since the GYS1 gene is knocked out in both astrocytes and neurons in these animals, it was not possible to determine the contribution of each cell type to these abnormalities.

Studying glycogen in the brain is challenging due to the rapid degradation of this polysaccharide after the interruption of brain circulation, which inevitably results in the loss of glycogen during histologic preparation of tissue even when perfusion of fixatives is used. Traditionally, it has been considered that brain glycogen is present only in astrocytes, where it is easy to identify thanks to its relatively high concentration (Cataldo and Broadwell, 1986; Hertz and Chen, 2018). However, the presence of the polysaccharide in specific subsets of neurons (e.g., motoneurons) has also been reported (Cammermeyer and Fenton, 1981; Borke and Nau, 1984). The most thorough analysis performed to date of the localization of brain glycogen in microwave-fixed brains (which stops enzymatic activity immediately and thus preserves the metabolic state) indicates that neurons may also contain glycogen, although at much lower concentrations than astrocytes (Oe et al., 2016). The notion of an active glycogen metabolism in neurons is supported by a key observation, namely the accumulation of glycogen in several pathological conditions like Lafora disease and Pompe disease (Sidman et al., 2008; Duran et al., 2014). However, this observation does not shed any light on the physiological role of the polysaccharide in these cells. In this regard, we previously demonstrated that primary cultured neurons have an active glycogen metabolism that protects them from hypoxia (Saez et al., 2014). However, since primary cultured neurons have embryonic features, the importance of neuronal glycogen in adult brains could differ to that in *in vitro* conditions.

To address the physiological role of neuronal glycogen, we generated a novel mouse model depleted of glycogen only in neurons. To this end, we used Camk2a Cre to delete GYS1 specifically in Camk2a neurons of the forebrain, including those of the CA1 pyramidal cell layer of the hippocampus, thus generating GYS1^Camk2a–KO^ mice. We selected these neurons since they are involved in memory and learning processes and participate in the hippocampal CA3-CA1 synapse, whose function can be analyzed electrophysiologically (Gruart et al., 2006). Although initially reported to occur exclusively in CA1 pyramidal neurons (Tsien et al., 1996), Cre recombinase-driven gene edition in Camk2A-Cre mice takes place in other pyramidal neurons of the forebrain and cortex (Victoria et al., 2016), which could thus contribute to the electrophysiological and learning deficits observed in GYS1^Camk2a–KO^ animals – these mostly related to prefrontal functions related to the acquisition of instrumental tasks (Jurado-Parras et al., 2012; Hasan et al., 2013) and to LTP induction in the intrinsic hippocampal circuit (Gruart et al., 2006).

Synaptic changes evoked by the presentation of a pair of pulses are commonly accepted as an indication of presynaptic short-term plasticity of hippocampal synapses, related to the process of neurotransmitter release (Zucker and Regehr, 2002). Thus, paired-pulse stimulation is used as an indirect measure of changes in the probability of neurotransmitter release at the presynaptic terminal (Fernandez De Sevilla et al., 2002; Zucker and Regehr, 2002; Lauri et al., 2007). Unlike GYS1^Nestin–KO^ mice (Duran et al., 2013), GYS1^Camk2a–KO^ animals did not show significant differences in paired-pulse facilitation with respect to their littermate controls. However, the trend may indicate a lower probability of neurotransmitter release. In contrast, postsynaptic LTP responses, a type of long-term synaptic plasticity, were significantly smaller in GYS1^Camk2a–KO^ mice. These results resemble those obtained in GYS1^Nestin–KO^ mice and indicate that neuronal glycogen participates in the correct establishment of LTP.

LTP has been proposed to be the mechanism underlying the acquisition of cognitive abilities at the cellular level. GYS1^Camk2a–KO^ animals showed a deficit in the acquisition of a complex associative task requiring inhibition of spontaneous exploratory activities. Again, although of a smaller magnitude, these results resemble those found in GYS1^Nestin–KO^ mice. Interestingly, fear conditioning was not affected in the neuron-specific KO mice, thereby indicating that other brain regions and circuits are not affected by the lack of glycogen in Camk2a neurons. Furthermore, and in contrast to GYS1^Nestin–KO^ mice, neuron-specific KO mice did not show greater susceptibility to hippocampal seizures or myoclonus induced by kainate administration or train stimulation. These results suggest that this alteration in GYS1^Nestin–KO^ mice is caused by the absence of astrocytic glycogen.

Interestingly, results collected during the operant conditioning task indicated that GYS1^Camk2a–KO^ mice reached the selected criterion for the fixed ratio (1:1) before control animals. We assumed that this was the result of hyperactive behavior, which was confirmed by the light/dark test. The light/dark test was designed to detect hyperactive or compulsive behaviors, and it has been successfully used in mice (Jurado-Parras et al., 2012; Hasan et al., 2013) and rats (Berger et al., 2017).

If glycogen in neurons is present at such low levels that make it very difficult to detect, then why is it important for brain function? Glycogen is mobilized very quickly and thus provides a faster way to obtain energy than through importing extracellular glucose. Furthermore, the product of glycogen breakdown is glucose-1P, which does not need the initial ATP required for glucose phosphorylation. Since the activity (and thus the energetic requirements) of neurons is pulsatile, a small amount of glycogen would be sufficient to cover this transient increase in energy needs. Furthermore, glycogen may be located in regions of high energetic demand, for example in synaptic boutons. If this were the case, the overall concentration of glycogen in neurons would still be low. In this regard, it is worth noting that glycogen molecules are small enough to fit into narrow processes in which mitochondria cannot. Furthermore, the activation of GS and consequent synthesis of glycogen during LTP induction could represent one of the mechanisms of synaptic plasticity.

In previous reports we showed that glycogen accumulation over a modest concentration is deleterious for neurons (Duran et al., 2012, 2014), thus raising the question as to why neurons have indeed maintained GS expression throughout evolution if its activation and the consequent deposition of glycogen cause the death of this cell population. This paradox led Magistretti and Allaman to refer to GS as a neuronal Trojan horse (Magistretti and Allaman, 2007). The observations described here clearly indicate that neurons do need an active glycogen metabolism to ensure proper functioning. Therefore, the challenge faced by neurons is how to maintain functionally active GS without allowing glycogen deposits to damage them.

In the light of the results presented here, we propose that neuronal glycogen is responsible for some of the roles previously attributed exclusively to astrocytic glycogen (e.g., experiments using glycogen degradation inhibitors to study the importance of brain glycogen in learning) (Hertz and Chen, 2018). Therefore, the relevance of neuronal glycogen for brain function should be reconsidered.

## Data Availability

All datasets generated for this study are included in the manuscript/supplementary files.

## Author Contributions

JD, JG, and JD-G conceived and designed the study. JD generated and bred the animal models. JD, OV, and IL-S performed the biochemical experiments and analyzed the data. AG and JD-G performed the electrophysiological and behavioral experiments, and analyzed the data. JD and JG wrote the first version of the manuscript. JD, OV, AG, and JD-G prepared the figures. All authors contributed to the improvement of the project and reviewed the final version of the manuscript.

## Conflict of Interest Statement

The authors declare that the research was conducted in the absence of any commercial or financial relationships that could be construed as a potential conflict of interest.
